# Supplementary material for: A Critical View of the Application of the APEX Software (Aqueous Photochemistry of Environmentally-Occurring Xenobiotics) to Predict Photoreaction Kinetics in Surface Freshwaters
Source: Molecules. 2019 Dec 18;25(1):9. doi: 10.3390/molecules25010009 (PMC7017383; doi:10.3390/molecules25010009)
Supplement: Supplementary file 1 [file molecules-25-00009-s001.zip › 3.2.4_gcc-4.4.0.zip!/3.2.4_gcc-4.4.0/3.2.4_gcc-4.4.0/doc/octave/html/interpreter/Character-Strings-in-Oct_002dFiles.html]

Character Strings in Oct-Files - Untitled


Next: Cell Arrays in Oct-Files,
Previous: Matrices and Arrays in Oct-Files,
Up: Oct-Files


---

#### A.1.3 Character Strings in Oct-Files

In Octave a character string is just a special `Array` class.
Consider the example

```
```
     #include <octave/oct.h>
     
     DEFUN_DLD (stringdemo, args, , "String Demo")
     {
       int nargin = args.length();
       octave_value_list retval; 
     
       if (nargin != 1)
         print_usage ();
       else
         {
           charMatrix ch = args(0).char_matrix_value ();
     
           if (! error_state)
             {
               if (args(0).is_sq_string ())
                 retval(1) = octave_value (ch, true);
               else
                 retval(1) = octave_value (ch, true, '\'');
     
               octave_idx_type nr = ch.rows();
               for (octave_idx_type i = 0; i < nr / 2; i++)
                 {
                   std::string tmp = ch.row_as_string (i);
                   ch.insert (ch.row_as_string(nr-i-1).c_str(), 
     			 i, 0);
                   ch.insert (tmp.c_str(), nr-i-1, 0);
                 }
               retval(0) = octave_value (ch, true);
             }
         }
       return retval;
     }
```
```

An example of the use of this function is

```
     s0 = ["First String"; "Second String"];
     [s1,s2] = stringdemo (s0)
     => s1 = Second String
             First String
     
     => s2 = First String
             Second String
     
     typeinfo (s2)
     => sq_string
     typeinfo (s1)
     => string
```

One additional complication of strings in Octave is the difference
between single quoted and double quoted strings. To find out if an
`octave_value` contains a single or double quoted string an example is

```
         if (args(0).is_sq_string ())
           octave_stdout <<
             "First argument is a singularly quoted string\n";
         else if (args(0).is_dq_string ())
           octave_stdout <<
             "First argument is a doubly quoted string\n";
```

Note however, that both types of strings are represented by the
`charNDArray` type, and so when assigning to an
`octave_value`, the type of string should be specified. For example

```
     octave_value_list retval;
     charNDArray c;
     ...
     // Create single quoted string
     retval(1) = octave_value (ch, true, '\'');
     
     // Create a double quoted string
     retval(0) = octave_value (ch, true);
```
